# Construction of a nomogram for predicting compensated cirrhosis with Wilson’s disease based on non-invasive indicators

**DOI:** 10.1186/s12880-024-01265-w

**Published:** 2024-04-16

**Authors:** Yan Li, Jing Ping Wang, Xiaoli Zhu

**Affiliations:** 1 Department of Ultrasound, The first affiliated hospital of Anhui University of Traditional Chinese Medicine, MeiShan Road, 230031 Anhui, P.R. China; 2 Department of Intervention, The First Affiliated Hospital of Soochow University, 899, The Pinghai Road, 215006 Jiangsu, P.R. China

**Keywords:** Wilson's disease, Cirrhosis, ARFI (acoustic radiation force impulse), Nomogram

## Abstract

**Background:**

Wilson’s disease (WD) often leads to liver fibrosis and cirrhosis, and early diagnosis of WD cirrhosis is essential. Currently, there are few non-invasive prediction models for WD cirrhosis. The purpose of this study is to non-invasively predict the occurrence risk of compensated WD cirrhosis based on ultrasound imaging features and clinical characteristics.

**Methods:**

A retrospective analysis of the clinical characteristics and ultrasound examination data of 102 WD patients from November 2018 to November 2020 was conducted. According to the staging system for WD liver involvement, the patients were divided into a cirrhosis group (*n* = 43) and a non-cirrhosis group (*n* = 59). Multivariable logistic regression analysis was used to identify independent influencing factors for WD cirrhosis. A nomogram for predicting WD cirrhosis was constructed using R analysis software, and validation of the model’s discrimination, calibration, and clinical applicability was completed. Due to the low incidence of WD and the small sample size, bootstrap internal sampling with 500 iterations was adopted for validation to prevent overfitting of the model.

**Results:**

Acoustic Radiation Force Impulse (ARFI), portal vein diameter (PVD), and serum albumin (ALB) are independent factors affecting WD cirrhosis. A nomogram for WD cirrhosis was constructed based on these factors. The area under the ROC curve (AUC) of the model’s predictive ability is 0.927 (95% CI: 0.88–0.978). As demonstrated by 500 Bootstrap internal sampling validations, the model has high discrimination and calibration. Clinical decision curve analysis shows that the model has high clinical practical value. ROC curve analysis of the model’s rationality indicates that the model’s AUC is greater than the AUC of using ALB, ARFI, and PVD alone.

**Conclusion:**

The nomogram model constructed based on ARFI, PVD, and ALB can serve as a non-invasive tool to effectively predict the risk of developing WD cirrhosis.

## Background

Wilson’s disease (WD) is an autosomal recessive genetic disorder. Although it has a low incidence rate, it primarily affects children and adolescents aged [1–3]. If WD fibrosis is not accurately diagnosed and properly treated, it may progress to cirrhosis and lead to severe complications such as decompensated cirrhosis with portal hypertension or liver failure.

Liver biopsy is considered the gold standard for fibrosis staging. However, the distribution of copper in WD patients is uneven, and the degree of liver fibrosis is also uneven, so liver biopsy cannot accurately represent the overall copper content and fibrosis stage of the whole liver [4–5]. Due to its invasiveness, high cost, potential complications, and ethical issues, liver biopsy is not the preferred method for assessing the degree of liver fibrosis. Therefore, non-invasive methods have become a research hotspot.

Expert consensus suggests that transient elastography (TE) is a reliable method for assessing liver fibrosis in viral hepatitis [6]. However, TE has limitations and may not provide accurate measurements for patients who are excessively obese, have ascites, or have a small rib cage aperture. Several studies have shown that acoustic radiation force impulse (ARFI) is more effective than TE in patients with ascites and obesity, and also has a lower failure rate [7]. ARFI has a sensitivity and specificity of 92% and 86%, respectively, for diagnosing cirrhosis [8]. ARFI has been successfully applied to liver diseases such as chronic hepatitis B and C, and can be recommended as a reliable tool for monitoring liver fibrosis [9–13]. Unfortunately, due to the rarity of WD, there is little research on the application of ARFI in WD.

This study aims to build a predictive model based on ultrasound imaging features and clinical characteristics to predict the risk of developing compensated WD cirrhosis. It evaluates and validates the feasibility and effectiveness of the model in diagnosing WD cirrhosis.

## Methods

### Patients

This retrospective study was conducted following the ethical principles of the Declaration of Helsinki and was approved by the hospital’s ethics review committee (registration number: 2021MCZQ02). The study initially included 122 patients diagnosed with Wilson’s disease (WD) from November 2018 to November 2020 based on inclusion criteria and finally included 102 cases after applying the following exclusion criteria. Demographic characteristics, complications, liver function biochemical indicators, copper-related biochemical indicators, zinc-related biochemical indicators, liver fibrosis index, routine ultrasound measurements, and ARFI were extracted from the electronic medical record system.

Inclusion criteria: (1) This study included patients diagnosed with WD, following the diagnostic guidelines for WD published by the European Association for the Study of the Liver (EASL) in 2012 [14]; (2) Age < 65 years; (3) Body mass index (BMI) range of 18.5∼28.0 kg/m²; (4) No history of other types of hepatitis or excessive alcohol consumption; (5) No rheumatic diseases that may lead to fibrosis; (6) No diabetes or renal failure.

Exclusion Criteria: Patients were excluded if they met any of the following criteria: (1) Incomplete ARFI examination or ultrasound diagnostic characteristic information; (2) Incomplete clinical biochemical examination data; (3) Poor quality of ARFI images in WD patients due to limb tremors.

**Fig. 1:**
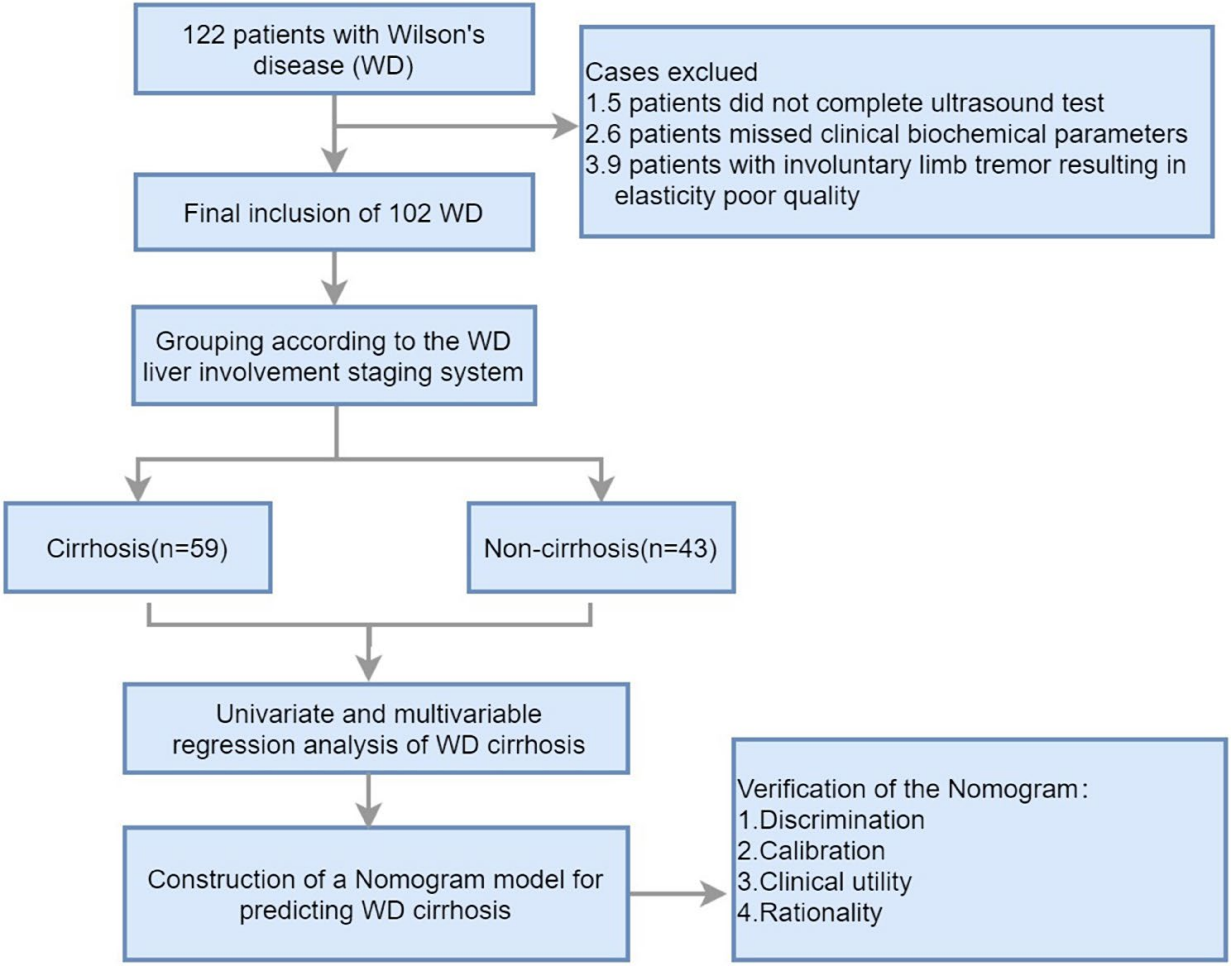
Flowchart illustrating the selection of the study population and the research

According to the inclusion criteria, a total of 122 WD patients were included in this study. However, 5 patients had incomplete ultrasound diagnostic information, 6 patients had incomplete clinical biochemical index data, and 9 patients had poor quality elastography images due to uncontrollable limb tremors. Therefore, these patients were excluded. Finally, the study population consisted of 102 patients. Figure 1 is a flowchart of the case screening and research process.

### Demographic and laboratory indexes

On the day of the ultrasound measurement, collect the patient’s demographic characteristics and medical history. Laboratory tests included alanine aminotransferase (ALT), aspartate aminotransferase (AST), serum albumin (ALB), prothrombin time (PT), serum copper, ceruloplasmin (CP), serum zinc, 24-hour urinary copper, 24-hour urinary zinc, PLT (platelet count), laminin (LN), hyaluronic acid (HA), collagen IV (CIV), pro-collagen protein III (P III N-P).

### The examination of conventional ultrasound

**Fig. 2:**
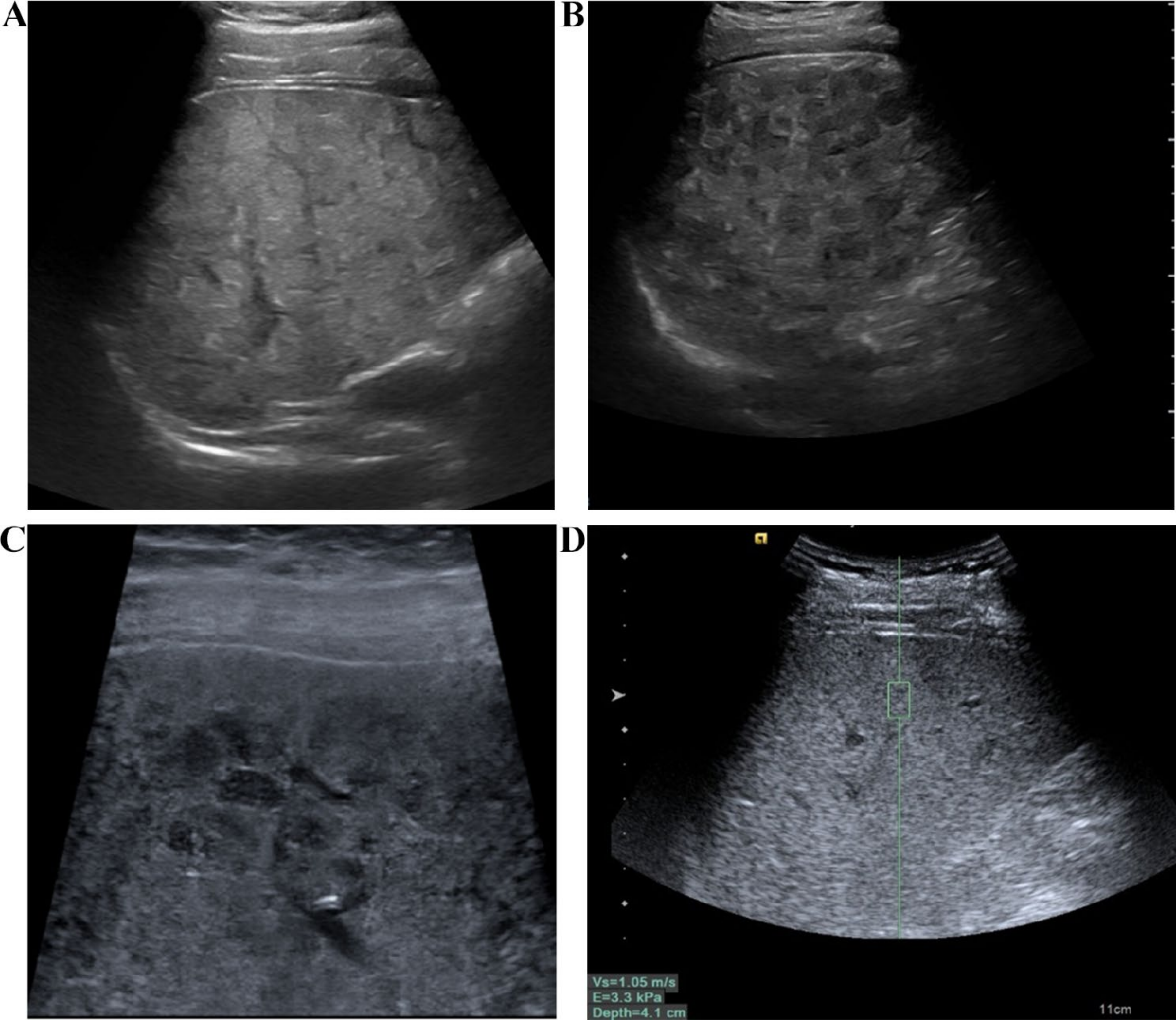
The examination of conventional ultrasound and ARFI. **A** The liver of patients with WD exhibited a patchy, fatty appearance. **B** Scattered nodular hypoechoic regions were observed in the liver of WD patients. **C** The liver of WD patients displayed honeycomb-like nodules when observed with a linear array probe, while the liver capsule appeared smooth. **D** Liver stiffness was assessed using ARFI technology

The instrument used is the Siemens Ultrasound System S2000, which is equipped with a convex array probe 4C1 (1–4 MHz) and a linear array probe 9L4 (4–9 MHz). After fasting for 8 h, the patient underwent a routine B-mode ultrasound examination. First, the entire liver was scanned using the low-frequency convex array probe (Fig. 2A, B), recording the morphological characteristics of the liver, portal vein velocity (PVV), portal vein diameter (PVD), spleen size, and ascites. When measuring the portal vein, the ultrasound beam was required to be as perpendicular to the portal vein as possible, measuring the maximum internal diameter of the portal vein. Take the median of six valid measurements as the final result. Then, the detailed characteristics of the liver capsule were examined using the high-frequency linear array probe (Fig. 2C).

### The measurements of ARFI

The ARFI examinations were conducted by two experienced ultrasound doctors with at least 10 years of abdominal ultrasound diagnostic experience. The ultrasound diagnosis was made by the two doctors, and a consensus on the diagnosis results was reached.

The instrument used is the Siemens Ultrasound System S2000, with a 4C1 probe and a frequency of 4.5 MHz. The instrument is equipped with Acoustic Radiation Force Imaging Virtual Touch Tissue Quantification (ARFI-VTQ) imaging mode.

The patient is placed in a supine position, lifting the right arm to open the intercostal space, and maintaining steady breathing. First, the patient underwent a routine ultrasound examination, documenting information such as the size and shape of the liver, the characteristics of the liver echo (Fig. 2A, B, C), the diameter and flow velocity in the portal vein, the size of the spleen, and any ascites. Then, the operator selects the S5 and S8 segments of the patient’s liver, with the image depth set at 4–6 cm. When the appropriate liver parenchyma ultrasound section (with fewer blood vessels and clear liver parenchyma) is selected, instruct the patient to hold their breath. When the ultrasound image on the display is still, the trigger key is pressed to capture the image. During measurement, the sampling frame is placed perpendicular to the liver capsule, at least 2–3 cm away from the liver capsule, avoiding large blood vessels and intrahepatic bile ducts (Fig. 2D). At the same sampling location, 10 sets of values are measured, with at least 6 sets being valid, and the median value is taken.

Quality Control: The IQR/Median (Interquartile Range/Median) ratio ≤ 0.3 is considered the reference for the quality control standard of this technique. During the examination of each patient, ARFI measurements should be taken from the same sampling section and position as much as possible.

### Staging of WD liver involvement

The staging system for WD liver involvement is as follows [15, 16]:

(I) Normal - no signs of laboratory or clinical abnormalities. (II) Elevated ALT with normal liver morphology features. (III) Abnormal liver morphology without cirrhotic manifestations. (IV) Clinical and imaging signs suggestive of compensated cirrhosis (Child-Pugh A). Compensated cirrhosis is diagnosed based on clinical manifestations, medical history, blood biochemistry, and imaging. The final diagnosis of compensated cirrhosis should meet two or more of the following criteria: ① Esophageal and gastric varices identified by endoscopy. ② Ultrasound, CT, and MRI are suggestive of cirrhosis or portal hypertension. ③ Hypersplenism characterized by thrombocytopenia (< 100 × 10^9^/L), after excluding other possible causes. ④ Decreased hepatic synthetic function characterized by prolonged prothrombin time (> 13 s) more than 7 days after discontinuation of thrombolytic or anticoagulant drugs, excluding other causes such as malnutrition or renal disease and decreased serum albumin (< 35 g/l). (V) Decompensated hepatic function (Child-Pugh B and C) or severely impaired cirrhosis. Decompensated cirrhosis is often accompanied by ascites, gastrointestinal bleeding, and hepatic encephalopathy. Combined with history, laboratory findings, and typical portal hypertension manifestations, decompensated cirrhosis can be easily diagnosed.

### Data analysis and statistical methods

Statistical analysis was conducted using SPSS 26.0 software and R (R4.1.3). Continuous variables with a normal distribution were presented as mean ± standard deviation (x̄±s), while those with a non-normal distribution were presented as median (interquartile range). Categorical data were presented as counts or percentages. For the comparison of normally distributed data between two groups, two independent sample t-tests were used. For non-normally distributed data, non-parametric Mann-Whitney U tests were employed. The Pearson chi-square test was used to compare categorical data between two groups. In case the conditions for the Pearson chi-square test were not met, either continuous corrected chi-square or Fisher’s exact test were used. Baseline description and analysis of differences were performed using the Compare Groups package in R. Multivariable logistic regression was conducted using the glm package in R. Discrimination analysis was performed using the pROC, ggROC, and fbroc packages in the R language. Calibration analysis was conducted using the calibrate function in the rms package in R, as well as the val. prob and HL test packages. Clinical decision curves were created using the rmda and dcurves packages in R, and nomograms were constructed using the rms package in R. To prevent overfitting in column line plots, bootstrap resampling was performed 500 times to evaluate the internal validation of predictive efficiency. The significance level was set at *p* = 0.05.

## Results

### Characteristics of patients

According to the staging system for liver involvement in WD, patients were divided into two groups: the WD cirrhosis group (WD liver involvement stage ≥ IV) and the non-cirrhosis group (WD liver involvement stage: I∼III). Table 1 shows the differences in demographic characteristics, complications, liver function biochemical indicators, copper-related biochemical indicators, zinc-related biochemical indicators, liver fibrosis index, routine ultrasound measurements, and ARFI between the two groups. The results revealed statistically significant differences between the groups in terms of age, ascites, ALB, PT, AST, PLT, PVV, PVD and ARFI. However, Other indicators showed no statistically significant difference between groups.

**Table 1:** Demographic and clinical characteristics of the study population

Characteristics	Total(*N* = 102)	Non-cirrhosis(*N* = 59)	cirrhosis(*N* = 43)	p
Age (Year)	28.5 (10.5)	26.5 (10.1)	31.2 (10.6)	0.024
Male/female(N)	62/40	33/26	29/14	0.332
**Spleen**				
Normal/ splenomegaly (N)	44 /58	26 /33	18/25	0.984
**Ascites**				
-/+(N)	91/11	58/1	33/10	<0.001
AST (U/L)	24.0 (20.0, 31.8)	23.0 (18.5, 29.5)	28.0 (22.0, 35.0)	0.034
ALT (U/L)	22.5 (17.0, 36.0)	21.0 (16.0, 41.5)	24.0 (17.0, 35.0)	0.551
PLT (x10^9^/L)	148 (106, 201)	166 (123, 200)	115 (52.5, 218)	0.007
ALB (g/l)	39.8 (36.8, 42.2)	40.8 (38.3, 43.0)	37.5 (32.3, 40.2)	<0.001
PT (sec)	11.4(10.7, 12.5)	11.1(10.4, 11.7)	11.9(11.2, 14.0)	< 0.001
Serum copper (umol/L)	4.00 (2.13, 5.97)	4.00 (2.08, 5.85)	4.06 (2.50, 6.65)	0.456
CP (g/L)	0.09 (0.06, 0.10)	0.09 (0.06, 0.10)	0.09 (0.05, 0.10)	0.904
Copper oxidase (/L)	0.02 (0.02, 0.04)	0.02 (0.02, 0.03)	0.02 (0.02, 0.05)	0.082
24 h urine copper (ug/24 h)	785 (499, 1263)	772 (480, 1262)	809 (611, 1288)	0.560
Serum zinc (umol/L)	20.6 (13.3, 72.9)	20.9 (13.5, 81.3)	18.0 (12.5, 60.0)	0.403
24 h urine zinc(ug/24 h)	3132 (1947, 5059)	3175(1936,4500)	2996(2062,5796)	0.792
LN (ng/ml)	86.0 (53.0, 104)	84.0 (59.3, 102)	90.0 (53.0, 104)	0.714
HA (ng/ml)	78.1 (53.0, 116)	78.2 (51.8, 135)	76.7 (53.0, 96.6)	0.412
CIV (ng/ml)	48.2 (31.9, 57.6)	51.7 (31.9, 68.7)	41.8 (21.8, 57.0)	0.089
P III N-P (ng/ml)	10.6 (8.01, 14.0)	10.4 (7.89, 13.9)	10.6 (8.56, 14.2)	0.402
PVV (cm/s)	17.1 (15.3, 19.1)	17.5 (16.3, 19.9)	16.0 (14.3, 18.5)	0.009
PVD (mm)	12.0 (10.0, 13.0)	11.0 (9.00, 12.0)	13.0 (11.5, 15.0)	<0.001
ARFI (m/s)	1.85 (0.35)	1.67 (0.24)	2.09 (0.33)	<0.001

*Note* Gender, splenomegaly, and ascites in the table are categorical variables, with data being count information, representing the number of patient cases. Non-normally distributed data are expressed as median (interquartile range) (M(IQR)), while normally distributed data are expressed as mean ± standard deviation (x̄±s). The remaining indicators are quantitative data. ALB (albumin), PT (prothrombin time), PVV (portal vein velocity), PVD (portal vein diameter), AST (aspartate aminotransferase), ALT (glutamic-pyruvic transaminase), CP (ceruloplasmin), PLT (platelets), LN (laminin), HA (hyaluronic acid), CIV (collagen IV), P III N-P (pro-collagen protein III)

### Univariate and multivariable regression analysis of WD cirrhosis and construction of the nomogram model

The assignment of classified variables in Logistic Regression was as follows: Ascites (+) = 1, Ascites (-) = 0, Splenomegaly (+) = 1, Normal Spleen Size = 0, Male = 1, Female = 0. Other indicators in the table were quantitative data and were input by original values. In the univariate regression analysis, factors with a p-value less than 0.1 were included in the multivariable regression analysis.

The results of the multivariable regression analysis indicated that ARFI and PVV were positively correlated with the risk of developing WD cirrhosis, with p-values less than 0.05 and regression coefficients of 6.1 and 0.44, respectively. ALB was negatively correlated with the risk of WD cirrhosis, also with a p-value less than 0.05 and a regression coefficient of -0.28 (Table 2). Other factors listed in the table did not show a significant correlation with the risk of cirrhosis, as their p-values were greater than 0.05. ARFI, PVV, and ALB were identified as independent predictors of WD cirrhosis.

**Table 2:** Univariate and multivariable regression analysis of WD cirrhosis

Characteristics	Univariate				multivariable			
	B	OR	95%CI	P	B	OR	95%CI	P
Gender	0.49	1.632	0.726–3.764	0.241				
Age	0.045	1.046	1.007–1.091	0.026				
CP	0.303	1.354	0-10822	0.957				
Copper oxidase	6.607	740.4	1.697–53,414	0.065				
Serum copper	0.066	1.068	0.923–1.239	0.376				
24 h urine copper	< 0.001	1	1-1.001	0.47				
24 h urine zinc	< 0.001	1	1–1	0.422				
Serum zinc	-0.005	0.995	0.982–1.006	0.372				
AST	0.013	1.013	0.985–1.046	0.371				
ALT	-0.001	0.999	0.985–1.011	0.824				
PT	-0.004	0.996	0.991–1.001	0.101				
ALB	-0.209	0.811	0.719–0.893	< 0.001	-0.28	0.75	0.621–0.872	0.001
PT	0.557	1.746	1.325–2.472	< 0.001				
PIIIN-P	0.066	1.069	0.99–1.166	0.104				
LN	< 0.001	1	1–1	0.779				
HA	< 0.001	1	0.999-1	0.468				
CIV	-0.004	0.996	0.985–1.005	0.419				
Ascites	18.147	7	0–10	0.988				
Splenomegaly	0.09	1.094	0.495–2.438	0.824				
PVD	0.361	1.435	1.19–1.771	< 0.001	0.44	1.55	1.177–2.145	0.004
PVV	-0.181	0.835	0.722–0.948	0.009				
ARFI	5.461	235.3	31.69–292	< 0.001	6.10	444	37.59–470	< 0.001

*Note* CP (ceruloplasmin), AST (Aspertate Aminotransferase), ALT (glutamic-pyruvic transaminase), PT (prothrombin time), ALB (albumin), PT (prothrombin time), P III N-P (pro-collagen protein III), LN (laminin), HA (hyaluronic acid), CIV (collagen IV), PVD (portal vein diameter), PVV (portal vein velocity)

**Fig. 3:**
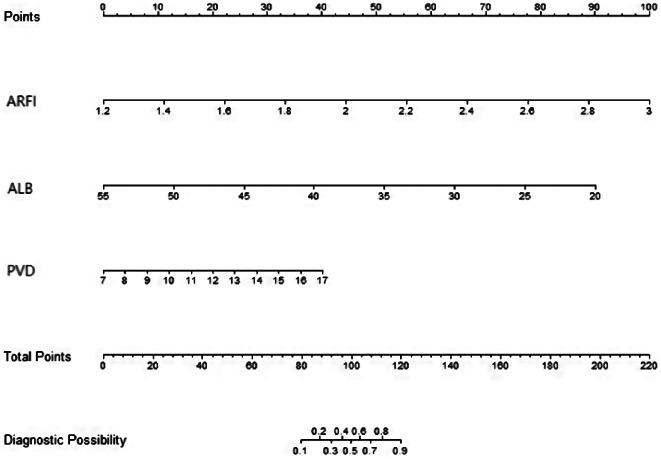
Construction of a nomogram model for predicting cirrhosis in WD based on ultrasound elastography, serum albumin, and ultrasound measurement of portal vein diameter. Note: ARFI (acoustic radiation force impulse), ALB (albumin), PVD (portal vein diameter)

A nomogram model was constructed to predict WD cirrhosis based on these factors (Fig. 3). In this nomogram, the value of each variable was projected onto the scale at the top to obtain the corresponding score. The scores of all variables were then summed to obtain the total score, which was compared to the total scoreline to determine the final predictive probability.

### Verification and evaluation of the prediction model

#### Discriminating ability of the model

**Fig. 4:**
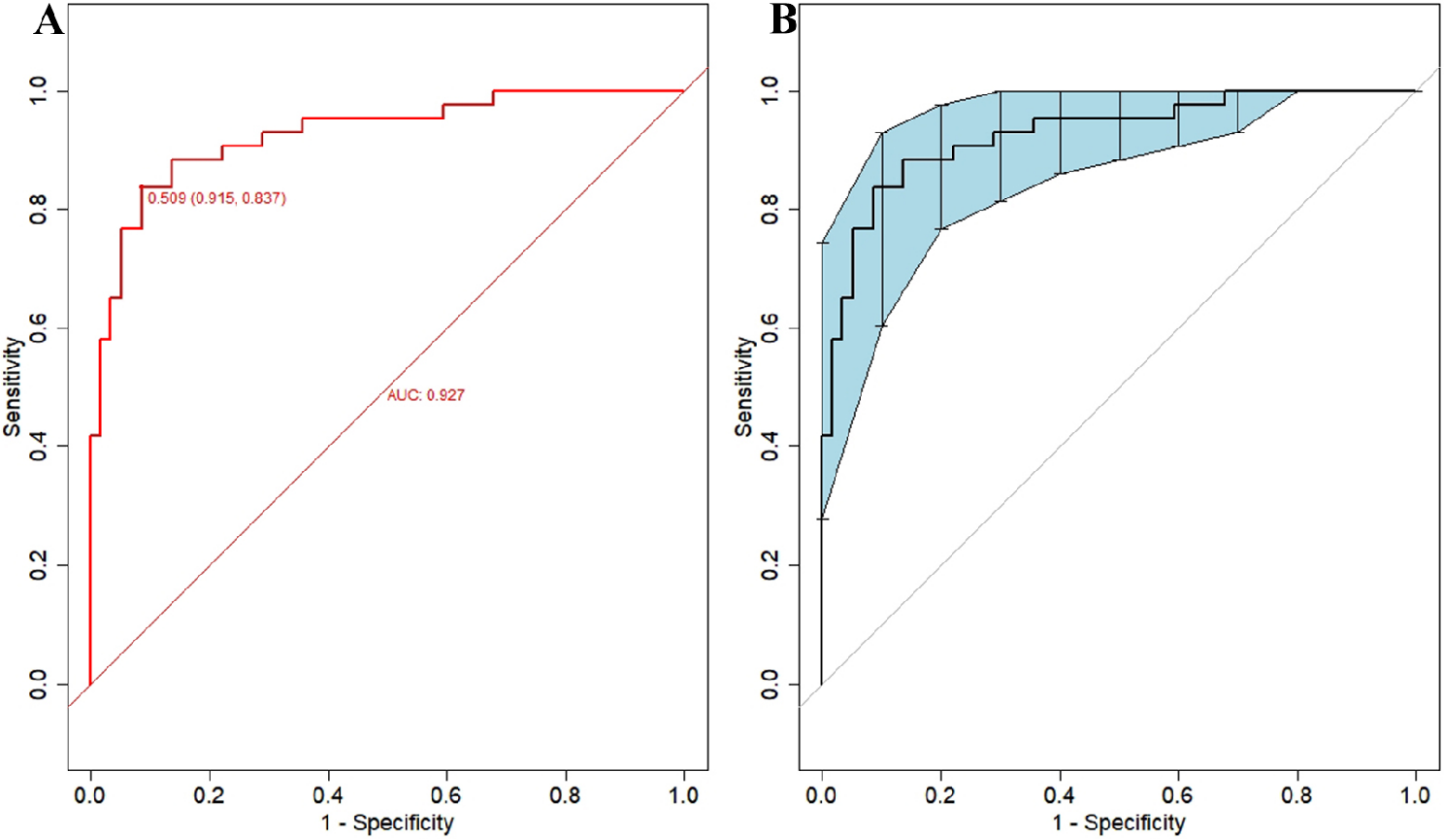
Discrimination of the nomogram model for predicting WD cirrhosis. *Note***A** Presented the ROC curve characteristics of the prediction model for diagnosing WD cirrhosis, with an area under the curve of 0.927 (95% CI: 0.88–0.978). **B** Depicted the ROC curve analysis of the model’s stability, which was validated through 500 iterations of bootstrap internal sampling, with a 95% CI of 0.869–0.97

Figure 4 illustrates the effectiveness of the model in distinguishing hepatic cirrhosis in Wilson’s disease. In the modeling dataset, the area under the receiver operating characteristic (ROC) curve for the predictive ability of the model was 0.927 (95% CI: 0.88–0.978), as depicted in Fig. 4A. The model’s predictive performance and stability were further validated through 500 iterations of bootstrap internal sampling, with a 95% CI of 0.869–0.97, as shown in Fig. 4B. The results indicated that this model exhibits excellent discrimination and a strong ability to distinguish hepatic cirrhosis in Wilson’s disease.

#### Calibration of the prediction model

**Fig. 5:**
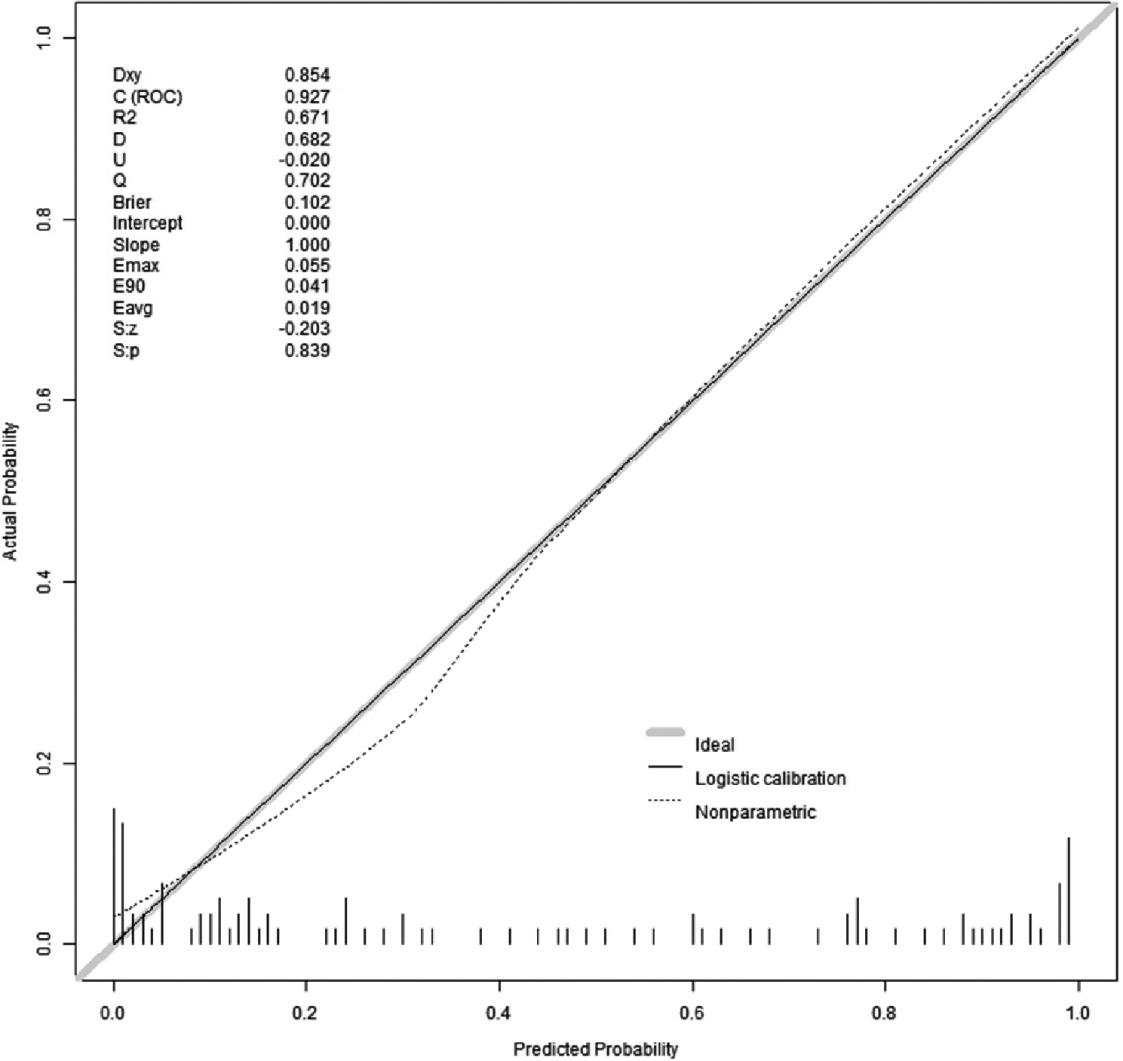
Calibration curves for model prediction of WD cirrhosis. Note: The model’s predicted probabilities were plotted on the x-axis, while the actual positive occurrence rate was plotted on the y-axis. The solid line (diagonal line) represented the ideal model, and the dashed line represented the calibration of the model established in this study. After conducting 500 iterations of bootstrap internal sampling, the model’s calibration analysis showed a Brier score of 0.102 and a p-value of 0.839(>0.05). These results indicate a high level of consistency between the predicted and actual outcomes

The results of the Hosmer-Lemeshow goodness-of-fit test for the prediction model revealed a chi-square value of 5.15 with a p-value of 0.82. Since the p-value for the Hosmer-Lemeshow test was greater than 0.05, it indicated that the model was well-calibrated. The calibration analysis of the model, depicted in Fig. 5, showcased the model’s calibration after 500 iterations of Bootstrap internal sampling. The Brier score was 0.102, and the p-value was 0.839(>0.05). The calibration curve demonstrated a strong correlation between the predicted probabilities and the actual occurrences.

#### Analysis of the clinical utility and rationality of the prediction model

**Fig. 6:**
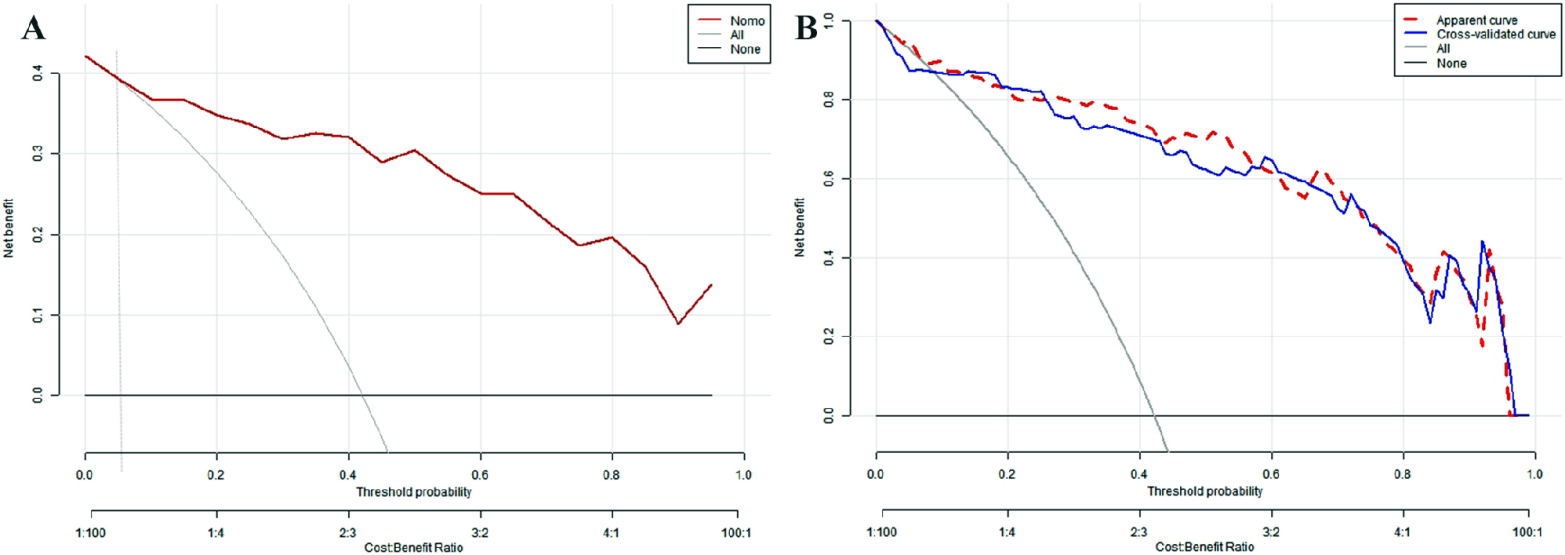
Clinical decision curve analysis of the model. *Note***A** is the decision curve of the model sampled by Bootstrap 500 times and **B** is the decision curve after cross-validation

To assess the clinical utility of the nomogram, we used the predicted probability from the calibration plot as the test variable and the occurrence of WD cirrhosis in patients as the state variable. We constructed a clinical decision curve (DCA) for the nomogram model, as shown in Fig. 6.

In the DSA curve, the two dashed lines represent the two extreme cases, with the grey horizontal line indicating that the model predicts there are no cirrhosis in all patients with WD and a clinical benefit of zero. The other grey line with a negative slope indicates that the model predicts there are cirrhosis in all WD patients, and the clinical benefit curve is a negative slope oblique line. The red curve represents the benefit for patients using the predictive model from this study. When the predicted probability is greater than the threshold of 0.05 (with a broader range), the red curve is higher than the grey horizontal line and the negatively sloped grey line, indicating that patients can benefit from the predictive model of this study.

**Fig. 7:**
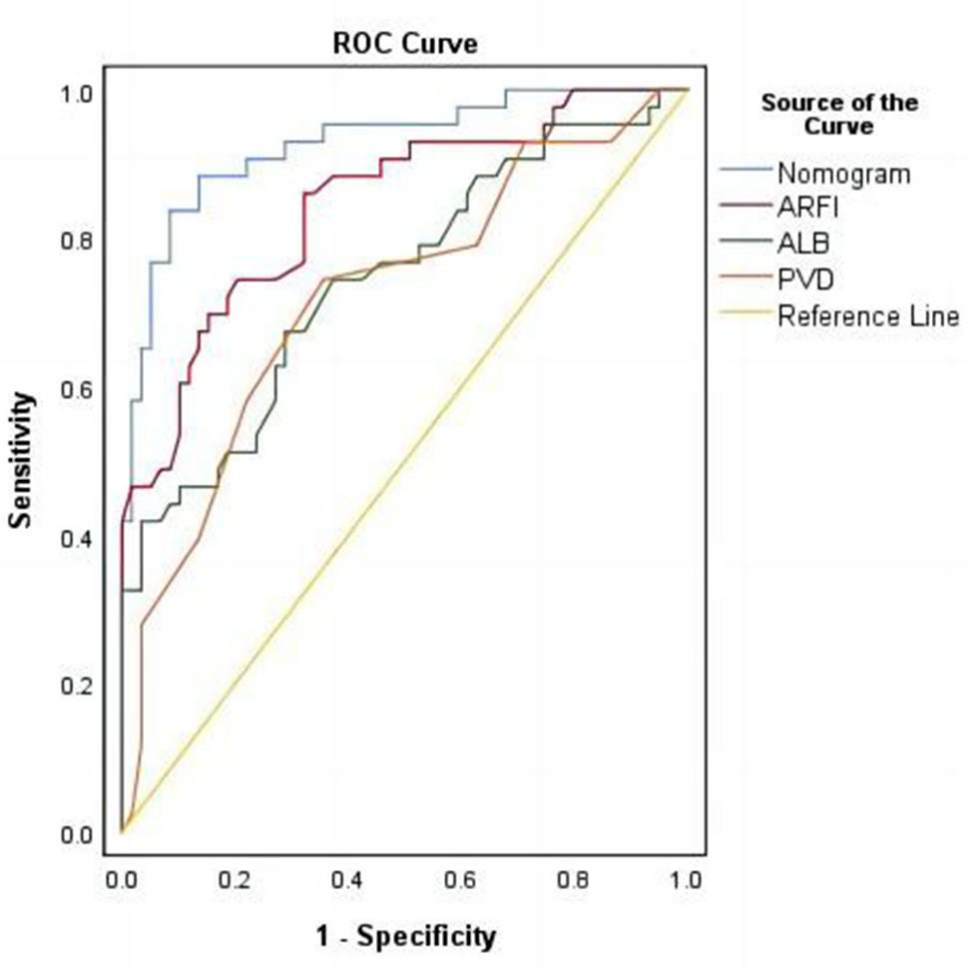
Reasonableness analysis of the predictive model (Nomo-ROC curve). *Note* The area under the curve for nomogram (model) is larger than the area under the curve for ALB, ARFI and PVD

Figure 7 demonstrated the rationality of the model, with the results indicating that the area under the curve for the nomogram was greater than the area under the curves for the individual use of ALB, ARFI, and PVD, this suggested that the ability to predict WD cirrhosis using this predictive model was superior to using ALB, ARFI, and PVD alone.

## Discussion

The liver plays a key role in maintaining copper metabolism balance. When this balance is disrupted, excessive copper accumulation in the liver can lead to liver damage, known as WD. Early changes of WD include hepatic steatosis, and over time, fibrosis around the portal vein progresses to cirrhosis [17–18]. Early diagnosis and timely treatment of cirrhosis are crucial for managing chronic liver diseases.

This study categorized WD patients into two groups: cirrhosis and non-cirrhosis, based on the criteria for WD liver involvement. The differences between the two groups were compared in terms of demographic characteristics, biochemical indicators of liver function, related biochemical indicators of copper and zinc, liver fibrosis index, routine ultrasound measurements, and ARFI. Indicators with significant differences between the groups were included in multivariable logistic regression analysis. Ultimately, the results showed that the independent influencing factors for WD cirrhosis were ALB, ARFI, and PVD, and a nomogram for WD cirrhosis was constructed. Since WD patients are rare, this study did not obtain external validation data from other research centers. However, the study used a method of 500 Bootstrap self-sampling for internal validation to prevent model overfitting. After validation of this model, it was shown to have high discriminative ability, calibration, clinical utility, and rationality.

### ARFI and PVD are important influencing factors of WD cirrhosis

ARFI technology is a type of ultrasound elastography technology. It uses focused ultrasonic beams as the excitation mechanism, causing longitudinal compression and lateral vibration in tissues when force is applied. This produces shear waves in the tissue, which are then captured using a specific electronic system to gather signals from the tissue. The propagation speed of shear waves in the region of interest can thus be obtained. The speed of shear waves is closely related to tissue elasticity [19]. By estimating the tissue elasticity modulus, one can indirectly reflect the degree of elasticity in that area. ARFI is similar to a physical palpation examination of the tissue, providing quantitative measurements of tissue hardness.

Several studies have shown a strong correlation between the results of ARFI (especially Siemens S2000) and liver biopsy results in various liver disease patients [20–22]. Currently, ARFI has established relatively standardized criteria for the diagnosis of viral hepatitis cirrhosis, but research on WD cirrhosis is limited, and diagnostic criteria have not yet been formulated. One guideline suggests using thresholds of 14.6 kPa and 10 kPa to diagnose or exclude hepatitis C cirrhosis [23]. A meta-analysis of non-invasive liver fibrosis assessment in patients with chronic hepatitis B and hepatitis C found that ARFI was accurate and reliable for diagnosing viral hepatitis liver fibrosis, and an ARFI value of 1.87 m/s could be used as the cutoff for significant liver fibrosis in hepatitis B [24], with ARFI values for cirrhosis being higher than those for significant liver fibrosis.

This study indicated that the threshold value for diagnosing WD cirrhosis was 1.88 m/s (E = 10.6 kPa). E represented the absolute value of the elastic modulus, and the conversion equation between E and Cs was: E = 3ρCs^2^, where ρ ≈ 1000 Kg/m^3^) [25–26], denoted tissue density, and Cs was the shear wave propagation speed in human tissues. The results showed that the threshold value for WD cirrhosis was lower than that for other liver disease cirrhosis. We analyzed the main reasons: (1) WD patients often exhibited thickening, enhancement, and nodular distribution along the portal vein in the liver parenchyma, which was related to higher copper deposition around the portal vein [27]. However, ARFI measurements should avoid the portal vein and were usually taken far from it, which may have led to an underestimation of LSM in WD patients. Secondly, WD typically began in childhood or adolescence and was treated early, leading to a stable course and relatively mild inflammation [28]. Thirdly, studies had shown that compared to hepatitis B, WD cirrhosis presented with less severe portal hypertension, lower prothrombin time and transaminase levels, and higher albumin levels [29]. Transaminases, albumin, and prothrombin time were closely related to the degree of liver inflammation, while liver stiffness was associated with liver fibrosis, inflammation, and portal pressure. Therefore, the inflammatory state of the liver and the pressure of the portal vein could affect LSM measurements, thus, it may affect the accuracy of liver fibrosis assessment [30–31].

In this study, another ultrasonographic indicator that affected WD cirrhosis was the PVD. Ultrasound measurement of the PVD was a non-invasive, simple, and reproducible method, usually used to assess liver blood flow and portal vein pressure. WD cirrhosis was caused by copper deposition in the liver, leading to hepatocyte degeneration, necrosis, fibrosis, and sinusoidal dilation. These pathological changes affected the histological structure of the liver tissue, thereby affecting blood reflux through the portal vein, resulting in an enlarged portal vein diameter. Studies have shown that ultrasound measurement of the PVD had high sensitivity and specificity for diagnosing WD cirrhosis, and the degree of liver inflammation or fibrosis was directly proportional to the width of the PVD [32]. Changes in the PVD indirectly reflected the degree of cirrhosis and changes in disease conditions.

### The value of the nomogram

In our previous study [33], we only conducted a multivariable regression analysis of the factors influencing WD cirrhosis, but multivariable logistic regression can only analyze the factors affecting positive events and cannot easily predict the probability of positive events. This study established a nomogram model based on our preliminary research. A nomogram can transform complex regression equations into visual graphics, characterized by being simple and easy to understand. Moreover, nomograms have strong visualization and operability. In clinical practice, projecting patients’ PVD, ARFI, and ALB measurement values onto the ruler at the top of the nomogram yields corresponding scores. Adding up all variable scores to obtain the total score, then comparing this score with the total score line, assesses the final predicted probability.

This article’s predictive model construction is based on the R data analysis system, which provides a unified framework for establishing predictive models, while machine learning often offers a variety of algorithms [34–35]. Currently, radiomics is a medical field with broad application prospects [36–37], and in future research on WD cirrhosis, we hope to explore radiomics analysis methods based on WD ultrasound images to further improve the accuracy of predictions.

This study has some limitations. First, it is a retrospective study, and the ideal study design should be a randomized controlled trial. Second, due to the rarity of WD, our research institution’s sample size is not large enough. In addition, the low incidence of WD makes it difficult to obtain relevant data from a large number of external research units, and models without external validation may lead to overfitting of the model, reducing its clinical applicability. However, this problem was partially resolved through internal validation by using 500 Bootstrap independent sampling. Another limitation is liver biopsy, which is the gold standard for diagnosing WD cirrhosis, but is not widely accepted in clinical practice. Therefore, this study could not obtain sufficient pathological reference but used the WD liver involvement staging system as the staging standard. However, this staging system has been cited in multiple studies related to WD.

## Conclusion

ARFI, PVD, and ALB are influencing factors in predicting WD cirrhosis. The nomogram model based on these three factors demonstrates high reliability and clinical utility. It can serve as a visual assessment tool for predicting the risk of WD cirrhosis, offering a convenient and user-friendly method.

## Data Availability

The data that support the findings of this study are available from the corresponding author, upon reasonable request.
